# Dendritic patch-clamp recordings from cerebellar granule cells demonstrate electrotonic compactness

**DOI:** 10.3389/fncel.2015.00093

**Published:** 2015-03-19

**Authors:** Igor Delvendahl, Isabelle Straub, Stefan Hallermann

**Affiliations:** Medical Faculty, Carl-Ludwig Institute for Physiology, University of LeipzigLeipzig, Germany

**Keywords:** granule cell, dendrites, cerebellum, patch-clamp techniques, electrophysiology

## Abstract

Cerebellar granule cells (GCs), the smallest neurons in the brain, have on average four short dendrites that receive high-frequency mossy fiber inputs conveying sensory information. The short length of the dendrites suggests that GCs are electrotonically compact allowing unfiltered integration of dendritic inputs. The small average diameter of the dendrites (~0.7 µm), however, argues for dendritic filtering. Previous studies based on somatic recordings and modeling indicated that GCs are electrotonically extremely compact. Here, we performed patch-clamp recordings from GC dendrites in acute brain slices of mice to directly analyze the electrotonic properties of GCs. Strikingly, the input resistance did not differ significantly between dendrites and somata of GCs. Furthermore, spontaneous excitatory postsynaptic potentials (EPSP) were similar in amplitude at dendritic and somatic recording sites. From the dendritic and somatic input resistances we determined parameters characterizing the electrotonic compactness of GCs. These data directly demonstrate that cerebellar GCs are electrotonically compact and thus ideally suited for efficient high-frequency information transfer.

## Introduction

Synaptic information transfer is strongly determined by the electrotonic properties of the postsynaptic neuron and the location of the synapse within the neuron. Dendrites receiving synaptic input provide the backbone for the computation performed by neurons (Magee, 2000; Abbott and Regehr, 2004; Gulledge et al., 2005; London and Häusser, 2005; Spruston, 2008). The morphology and passive properties of dendrites critically influence the processing of synaptic inputs (Jack et al., 1983; Mainen and Sejnowski, 1996; Segev and London, 2000; Schaefer et al., 2003; Abrahamsson et al., 2012). Thus, knowledge about the electrical properties of dendrites is crucial for our understanding of information transfer and computation in the central nervous system.

Cerebellar granule cells (GCs) are the most numerous neurons in the brain (Williams and Herrup, 1988) and compose the majority of the input layer of the cerebellar cortex (Billings et al., 2014). GCs have small somata and, on average, four short dendrites (Palkovits et al., 1972; Palay and Chan-Palay, 1974). The dendrites end with claw-like shaped digits (DiGregorio et al., 2007), which receive excitatory mossy fiber input in cerebellar glomeruli (D’Angelo et al., 1990; Silver et al., 1992). A glomerulus is formed by a single presynaptic mossy fiber bouton, Golgi cell axons, and dendrites of more than 10 GCs (Jakab and Hámori, 1988; Billings et al., 2014; Ritzau-Jost et al., 2014). GCs integrate the broad-bandwidth sensory information conveyed by mossy fiber inputs, transforming it into higher dimensional, sparser code (Marr, 1969; Billings et al., 2014). Thus, the anatomical structure of the GC layer is optimal for pattern separation (Olshausen and Field, 2004), which is important for network functions such as adaptive filtering (Fujita, 1982; Dean et al., 2010) and associative learning (D’Angelo and De Zeeuw, 2009).

Regarding the electrical properties of cerebellar GCs, previous studies based on somatic recordings and modeling indicated that these small neurons are electrotonically compact (Silver et al., 1992; D’Angelo et al., 1993; Gabbiani et al., 1994), thus affording good somatic voltage-clamp. Consequently, GC soma and dendrites are generally assumed to form a single electrical compartment, thereby acting as a point neuron (Billings et al., 2014). In recent years, direct patch-clamp recordings from dendrites have significantly advanced our understanding of many neurons’ electrical properties and their signaling (see e.g., Stuart and Sakmann, 1994; Nevian et al., 2007; Hu et al., 2010). The electrotonic properties of the small GC dendrites, however, have not been directly determined. In particular, passive membrane properties of GC dendrites such as the input resistance and their relation to somatic values remain unclear. Furthermore, model predictions critically depend on the diameter of dendrites, which is difficult to measure. Here, we establish whole-cell patch-clamp recordings from GC dendrites to directly determine their electrotonic properties. We compare the input resistance and measure spontaneous excitatory postsynaptic potentials (EPSP) at dendritic and somatic recording sites. Our experimental findings provide direct evidence for the electrotonic compactness of GCs.

## Materials and Methods

### Electrophysiology

Cerebellar slices were prepared from mature (P37 ± 3, range P22–P98) CD-1 or C57BL/6 mice of either sex. Animals were bred in the animal facility of the Medical Faculty of the University of Leipzig, and treated in accordance with the German Protection of Animals Act (TierSchG §4 Abs. 3) and with the guidelines for the welfare of experimental animals issued by the European Communities Council Directive of 24. November 1986 (86/609/EEC). The local authorities approved the experiments (Landesdirektion Leipzig, registration number T86/13). Mice were housed in a 12 h light/dark cycle with food and water ad libitum. Animals were lightly anesthetized with isoflurane (Baxter, Deerfield, IL) before being killed by rapid decapitation. The cerebellar vermis was quickly removed and mounted in a chamber filled with chilled extracellular solution. Parasagittal 300-µm slices were cut using a Leica VT1200 microtome (Leica Microsystems, Wetzlar, Germany), transferred to an incubation chamber at ~35°C for 30 min and subsequently stored at room temperature. Artificial cerebrospinal fluid (ACSF) was used for slice cutting, storage, and experiments. ACSF contained (in mM): 125 NaCl, 25 NaHCO_3_, 2.5 KCl, 1.25 NaH_2_PO_4_, 2 CaCl_2_, 1 MgCl_2_, 20 Glucose (~310 mOsm, pH 7.3 when bubbled with Carbogen (5% O_2_/95% CO_2_)). Patch pipettes were pulled from borosilicate glass (Science Products, Hofheim, Germany) using a DMZ Puller (Zeitz-Instruments, Martinsried, Germany). Patch pipettes had open-tip resistances of 9–14 MΩ or 14–18 MΩ for somatic and dendritic recordings, respectively. The intracellular solution contained (in mM): 150 K-gluconate, 10 NaCl, 10 K-HEPES, 3 Mg-ATP, 0.3 Na-GTP (300–305 mOsm, pH adjusted to 7.3 with KOH). In addition, the intracellular solution contained 10–20 µM of the fluorescence dye Atto594. Experiments were performed at 35–37°C and slices were continuously superfused with ACSF. Atto594 was obtained from Atto-Tec (Atto-Tec, Siegen, Germany); all other chemicals were purchased from Sigma-Aldrich (St. Louis, MO).

**Figure 1:**
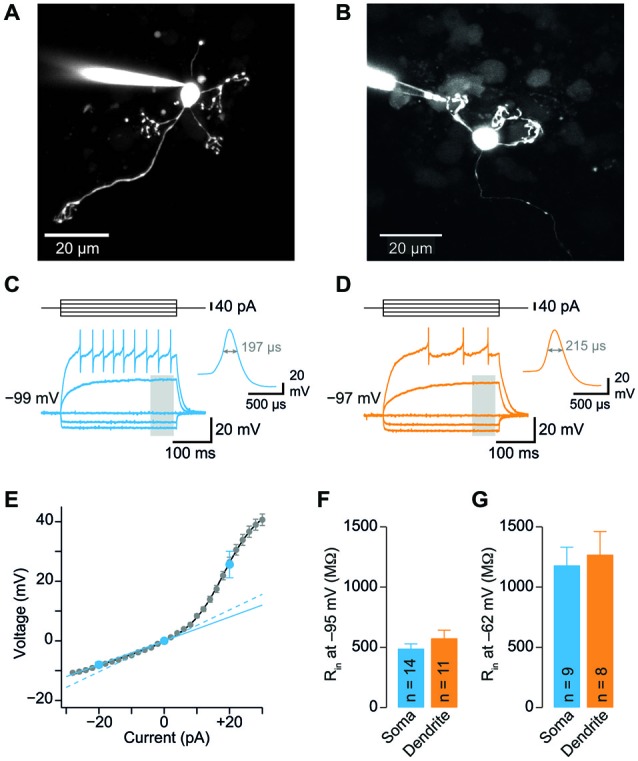
**Somatic and dendritic input resistances are similar. (A)** Cerebellar granule cell (GC) filled with a fluorescent dye via the patch-pipette during a somatic recording. Maximum intensity projection of a two-photon stack of 11 images, z-step 2 µm.** (B)** GC filled with a fluorescent dye via the patch-pipette during a dendritic recording. Maximum intensity projection of a two-photon stack of 8 images, z-step 3 µm.** (C)** Voltage transients obtained in response to tonic current injections (steps of ± 20 pA) in a somatic GC recording. Action potentials are truncated for clarity. Inset shows action potential on enlarged time scale, the duration at the half-maximal amplitude is indicated. Same cell as in **(A)**. The gray area indicates time window used for analysis of input resistance.** (D)** Corresponding data from a dendritic recording. Same cell as in **(B)**. **(E)** Voltage-current relation determined with small current steps in *n* = 35 GC somata in a different set of experiments (gray markers). Blue markers represent data obtained with ± 20 pA steps. Input resistance determined using small (2 pA, dashed line) and larger steps (−20 pA, continuous line) differed by a factor of 1.3. Input resistance measured using −20 pA steps was corrected by this factor (see *Material and Methods*). **(F)** Average input resistance (*R_in_*) at GC somata and dendrites was not significantly different. *R_in_* was determined at a membrane potential of −95.4 ± 1.1 mV (corresponding to 0 pA in panel **E**). Bars represent means ± SEM (number of somatic and dendritic GC recordings is indicated).** (G)** *R_in_* recorded from somata and dendrites at a membrane potential of −62.4 ± 3.2 mV (corresponding to +20 pA in panel **E**).

Cerebellar GCs were visualized with oblique infrared illumination and were identified as previously described (Silver et al., 1996). For dendritic recordings, putative cerebellar glomeruli were approached with patch-pipettes. In 12 out of >700 attempts, a whole-cell recording could be established at a GC dendrite. Dendritic recording sites were confirmed by two-photon imaging of the GC filled with Atto594 via the dendrite (Figure 1B). Patch-clamp recordings were made using a HEKA EPC10/2 USB amplifier (HEKA Elektronik, Lambrecht/Pfalz, Germany). Data were sampled at 200 kHz. Measurements were corrected for a liquid junction potential of +13 mV. Series resistance ranged from 20–57 MΩ for somatic recordings (mean 36.6 ± 2.4 MΩ), and from 42–155 MΩ for recordings from GC dendrites (mean 88.7 ± 16.9 MΩ).

### Two-photon Imaging

We used a Femto2D laser-scanning microscope (Femtonics, Budapest, Hungary) for imaging. Two-photon excitation was performed with a MaiTai femtosecond pulsed Ti:Sapphire laser (SpectraPhysics, Santa Clara, CA) tuned to 810 nm. Both reflected and transmitted fluorescence were collected by the imaging setup with a 60× water-immersion objective (Olympus, NA 1.0) and an oil-immersion condenser (Olympus, NA 1.4), respectively. Imaging data were acquired and processed using MES software (Femtonics). Stacks of two-photon images covering 20–50 µm in *z*-dimension were obtained. Diameters of GC dendrites and somata were measured as full-width at half-maximum of intensity line profiles made perpendicular to the dendrite or soma, respectively, in maximum *z*-projections of image stacks. Dendrite length was measured as *xyz*-distance in image stacks using MES software.

### Data Analysis

Input resistance (*R_in_*) was calculated from voltage deflections in response to tonic current injection (−20 pA, duration 300 ms). Voltage was calculated as mean over 60 ms at steady-state. In a separate set of experiments, the subthreshold current-voltage relationship was determined with small current steps (±2 pA) in order to characterize the dependence of *R_in_* on the amplitude of current injection. As previously reported (D’Angelo et al., 1995; Cathala et al., 2003), GCs exhibited outward and inward rectification (Figure 1E). Consequently, the data obtained with −20 pA current steps were corrected for by using the slope at 0 pA of a sum of a sigmoid and a linear function fit to the data, resulting in a correction factor of 1.3 (Figure 1E). Spontaneous EPSP were detected with a template matching routine implemented in NeuroMatic software.^1^ For analysis of 20–80% rise times and decay time constants of EPSPs, data were filtered to avoid distortions of the kinetics measurements by noise. Statistical analysis was performed using unpaired or paired *t*-tests. Level of statistical significance was set at *p* < 0.05. Data are expressed as mean ± SEM except where stated.

### Modeling

To determine the electrotonic properties of GCs from the somatic and dendritic input resistance, the following approach was used: GCs were represented by a spherical soma with radius, *a*_soma_, an axon with radius, *a*_axon_, and four cylindrical dendrites with radius, *a*_dend_ (Figure 3A). Dendritic claws were not included as additional compartments, because their diameter does not exceed the diameter of the parent dendrite (Jakab and Hámori, 1988; DiGregorio et al., 2002). The resulting somatic input conductance, *g*_somatic_, is:

(1) $$g_{\text{somatic}}=g_{\text{soma}}+4g_{\text{dendrite}}+g_{\text{axon}}$$

**Figure 2:**
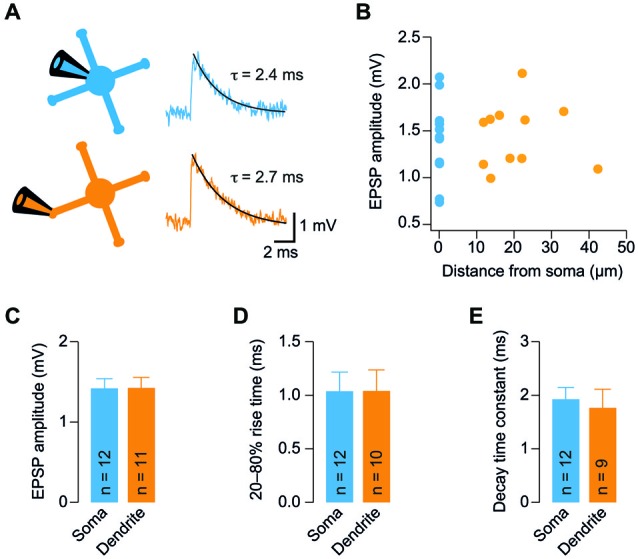
**GC dendrites do not significantly filter spontaneous EPSPs. (A)** Representative spontaneous excitatory postsynaptic potentials (EPSP) recorded from the soma (blue) or dendrite (orange) of GCs. Traces were digitally filtered to 8 kHz (−3 dB cut-off) for display. The recording configurations are illustrated on the left.** (B)** Dendritic EPSP amplitude as a function of distance from soma. For comparison, the somatically recorded EPSP amplitude is plotted in blue (*n* = 12 and *n* = 11 somatic and dendritic recordings, respectively).** (C)** EPSP amplitude was comparable at somatic and dendritic recording sites.** (D)** The 20–80% EPSP rise time did not differ significantly between soma and dendrites.** (E)** EPSP decay time constants were similar for somatic and dendritic recording sites. All bargraphs show means ± SEM (number of somatic and dendritic GC recordings is indicated).

**Figure 3:**
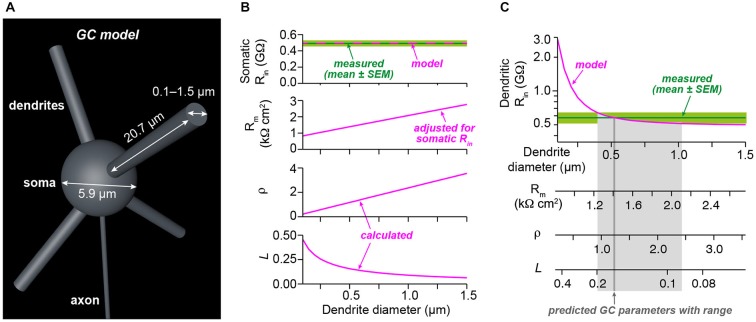
**Analysis of electrotonic properties demonstrates electrical compactness. (A)** Illustration of our GC model. The model consisted of a spherical soma and four dendrites. The indicated diameter of the soma and the length of dendrites were measured from stacks of two-photon microscopic images obtained during dendritic recordings. Axon diameter was taken from the literature. The diameter of the dendrites was systematically varied between 0.1 and 1.5 µm. **(B)** Superposition of the measured somatic input resistance (*R_in_*) with the prediction of the model. For each diameter of the dendrites, the specific membrane resistance (*R*_m_) was adjusted to ensure the correct somatic *R_in_*. The dendrite-to-soma conductance ratio (*ρ*) and the electrotonic length of the dendrites (*L*) were calculated as a function of dendrite diameter.** (C)** Superposition of the measured dendritic *R_in_* with the prediction of the model as a function of dendrite diameter. Comparison of the model prediction with the mean and the SEM of the dendritic *R_in_* revealed estimates with confidence ranges for the dendrite diameter, *R*_m_, *ρ*, and *L* (see Table 1).

**Table 1:** **Parameters of GCs**.

Parameter	Value	Method
Somatic *R_in_* at –95 mV (MΩ)	492 ± 37 (*n* = 14)	patch-clamp recording
Dendritic *R_in_* at –95 mV (MΩ)	578 ± 65 (*n* = 11)	patch-clamp recording
Somatic *R_in_* at –62 mV (MΩ)	1182 ± 150 (*n* = 9)	patch-clamp recording
Dendritic *R_in_* at –62 mV (MΩ)	1273 ± 189 (*n* = 8)	patch-clamp recording
Soma diameter (µm)	5.9 ± 0.3 (*n* = 11)	two-photon imaging
Dendrite length (µm)	20.7 ± 2.9 (*n* = 11)	two-photon imaging
Dendrite diameter (µm)	0.69 ± 0.3 (*n* = 10)	two-photon imaging
Dendrite diameter (µm)	0.52 (0.40–1.02)	**Figure 3C**
*R_m_* (kΩcm^2^)	1.42 (1.25–2.12)	**Figure 3C**
*ρ*	1.23 (0.93–2.43)	**Figure 3C**
*L*	0.15 (0.09–0.19)	**Figure 3C**

*Summary of determined GC parameters (mean ± SEM, or mean with 16–84% confidence interval in brackets). The input resistance (*R_in_*) was obtained with somatic or direct dendritic patch-clamp recordings. The diameter of the soma and the length of the dendrites were measured from stacks of two-photon images. The diameter of the dendrites, the specific membrane resistance (R_m_), the dendrite-to-soma conductance ratio (ρ) and the electrotonic length of the dendrites (L) were determined in Figure 3C*.

where *g_soma_*, *g_axon_*, and *g_dendrite_* are the input conductance of an isolated soma, isolated axon, and a single isolated dendrite, respectively. *g_soma_* is calculated as:

(2) $$g_{s\text{oma}}=\frac{1}{R_{m}}4\pi a_{s\text{oma}}^{2}$$

where *R_m_* is the specific membrane resistance. *g_dendrite_* is calculated as the input conductance of a finite cable (Rall, 1969; Jack et al., 1983):

(3) $$g_{\text{dendrite}}=\frac{\tanh L}{r_{a}\lambda }$$

where *L* is the electrotonic length of the dendrites, *r*_a_ is the intracellular resistance to axial flow of current along the cylinder, and λ is the membrane length constant defined as:

(4) $$L=\frac{l}{\lambda }$$

where *l* is the length of the dendrite,

(5) $$r_{a}=\frac{R_{i}}{\pi a_{\text{dend}}^{2}}$$

where *R*_i_ is the intracellular resistivity, and

(6) λ=Rmadend2Ri

g*axon* is calculated as the input conductance of a finite cable, accordingly.

First, *R*_m_ was calculated to obtain the measured somatic input resistance as a function of dendrite diameter (= 2*a*_dend_; Figure 3B) by numerically solving equation (1) for *R*_m_ (using the FindRoot function of Mathematica). Note, that the three remaining parameters were measured (*a*_soma_ and *l*) or taken from the literature (*R*_i_, Silver et al., 1992; Gabbiani et al., 1994; Cathala et al., 2003). In addition, two parameters describing the electrotonic compactness of neurons were plotted as a function of dendritic diameter: The above defined electrotonic length of the dendrites, *L*, and the dendrite-to-soma conductance ratio, *ρ* (also referred to as dendritic dominance), defined as (Rall, 1969; Jack et al., 1983):

(7) $$\rho =\frac{4g_{\text{dend}}}{g_{\text{soma}}}$$

Finally, the predicted dendritic input resistance was calculated using the NEURON simulation environment (Carnevale and Hines, 2006). A multi-compartment cylinder (*nseg* = 20) with radius *a*_soma_ and length 2*a*_soma_ represented the soma; the axon was represented by a multi-compartment cylinder (*nseg* = 20) with radius *a*_axon_ = 0.09 µm (Sultan, 2000) and length 300 µm. Increasing the length of the axon had marginal impact on the results. Four cylinders with radius *a*_dend_ and length *l* represented the dendrites (see Table 1; *nseg* = 20). Membrane capacitance (*C*_m_) was 0.9 µF cm^−2^. For each dendritic diameter, *R*_m_ of the NEURON model was set to a value ensuring the correct somatic input resistance. Current injection at the soma resulted in voltage deflections at the soma consistent with the calculated somatic input resistance. Current injection at the tip of one dendrite resulted in voltage deflections at the tip of the dendrite from which the dendritic input resistance was calculated.

## Results

### Somatic and Dendritic Input Resistances are Similar

To investigate the electrotonic properties of cerebellar GCs, we performed direct patch-clamp recordings from GC somata and dendrites (Figures 1A,B). For dendritic recordings, putative cerebellar glomeruli containing mossy fiber boutons and dendrites of GCs were approached with patch pipettes. After establishing the whole-cell configuration, GCs were unequivocally identified by the following two criteria: (1) In contrast to presynaptic mossy fiber terminals, which fire a single action potential upon current injection (Rancz et al., 2007; Ritzau-Jost et al., 2014), GCs display distinctive repetitive firing (Cathala et al., 2003); and (2) The dendritic recording site was verified by including a fluorescence dye (Atto594) in the patch pipette and using two-photon imaging (Figure 1B). Interestingly, in 5 out of 12 GCs the axon originated from the dendrite (Thome et al., 2014). In all our experiments, the dendritic recording site was located at the distal part of the dendrites with an average distance from the soma of 20.7 ± 2.9 µm (*n* = 11; range: 11–42 µm; Table 1). Thus, our data show that direct patch-clamp recordings from the small dendrites of cerebellar GCs are feasible.

We compared the input resistance (*R_in_*) of somatic and dendritic recordings to investigate the electrotonic compactness of GCs. Analysis of the spatial distribution of *R_in_* alone is necessary, but not sufficient to make conclusions on electrical compactness of neuronal structures. In our case, however, the length of dendrites is known, which allows investigating the electrical compactness of GCs with additional knowledge of *R_in_*. We determined *R_in_* in current-clamp mode using 300-ms long hyperpolarizing current steps of –20 pA (Figures 1C,D). Because *R_in_* depends on the amount of current injection (D’Angelo et al., 1995; Cathala et al., 2003), we also determined the voltage-current relation in a separate set of GC somatic recordings using smaller (±2 pA) current steps. These data were fit with the sum of a sigmoid and a linear function. From this fit, *R_in_* was determined as the slope at 0 pA, which was 1.3-fold higher than *R_in_* calculated from −20 pA step current injections (Figure 1E, blue lines). Therefore, *R_in_* values measured in somatic and dendritic recordings using −20 pA current injection were corrected accordingly (cf. *Material and Methods*). Interestingly, *R_in_* was not significantly different at dendritic and somatic recording sites (soma: 0.49 ± 0.04 GΩ; dendrite: 0.58 ± 0.07 GΩ; *p* = 0.24, unpaired *t-test*; Figure 1F; Table 1). Also, *R_in_* in our somatic measurements was comparable to values previously reported for P39 mice (Cathala et al., 2003) and adult cats (Jörntell and Ekerot, 2006), but lower than previously determined in young rats (D’Angelo et al., 1993, 1995; Silver et al., 1996; Prestori et al., 2013). Cerebellar GCs show pronounced inward rectification ((D’Angelo et al., 1995; Cathala et al., 2003), cf. Figures 1C,D,F), which could impact the *R_in_* measurements with hyperpolarizing current steps. When analyzing *R_in_* with depolarizing current steps of +20 pA, we obtained higher values, which were again similar in somatic and dendritic recordings (soma: 1.18 ± 0.15 GΩ; dendrite: 1.27 ± 0.19 GΩ, Figure 1G; Table 1). These data directly demonstrate that the distal part of the dendrites of GCs has similar *R_in_* compared to the soma. Furthermore, the membrane time constant (τ_m_) determined with hyperpolarizing current injections was comparable for somatic and dendritic recordings (soma: 1.4 ± 0.12 ms, dendrite: 1.63 ± 0.17 ms; *p* = 0.25, unpaired *t*-test).

### GC Dendrites do not Significantly Filter Spontaneous EPSPs

The similar *R_in_* of soma and dendrites suggests that GCs are electrotonically compact. To further investigate this hypothesis, we measured spontaneous EPSPs in GC somata and dendrites. We observed spontaneous EPSPs (Figure 2A) at a mean frequency of 0.85 ± 0.16 Hz and 1.19 ± 0.22 Hz in somatic and dendritic recordings, respectively, consistent with previous reports (Cathala et al., 2003; Hallermann et al., 2010). The amplitude of spontaneous EPSPs of GCs did not display a strong dependence on distance of dendritic recording sites from the soma (Figure 2B). Accordingly, the mean amplitude of spontaneous EPSPs was not significantly different between somatic and dendritic recording sites (*p* = 0.98, unpaired *t*-test; Figure 2C). In addition, the rise times and decay time constants of spontaneous EPSPs were comparable at the two distinct recording sites (20–80% rise time: 1.04 ± 0.18 ms vs. 1.04 ± 0.19 ms, *n* = 12 and 10, *p* = 0.99; decay time constant: 1.9 ± 0.2 ms vs. 1.8 ± 0.4 ms, *n* = 12 and 9, *p* = 0.68; for somatic and dendritic recordings, respectively, unpaired *t-tests*; Figures 2D,E). When recording from a dendrite, some EPSPs will be locally generated and the rest originate from the remaining three dendrites. However, we did not observe an increased heterogeneity of the EPSP amplitude during the dendritic recordings (dendritic vs. somatic coefficient of variation, CV = mean/SD: 50.4% vs. 61.4%) and the variability of kinetic parameters was comparable (20–80% rise time dendritic vs. somatic CV: 111.4% vs. 91.8%; decay time constant dendritic vs. somatic CV: 93.4% vs. 76.8%). These results indicate that dendrites do not filter EPSPs in GCs to a large extent.

### Analysis of Electrotonic Properties Demonstrates Electrical Compactness

We next determined the electrotonic properties of GCs by analyzing the measured somatic and dendritic input resistance using analytical calculations and numerical modeling implemented in NEURON (Carnevale and Hines, 2006). GCs were modeled by a spherical soma with a cylindrical axon and four cylindrical dendrites (Figure 3A). The somatic input resistance of this simplified GC can be calculated analytically (Equation 1, *Material and Methods*) and depends on the diameter of the soma, the length and diameter of the axon and dendrites, the intracellular resistivity, and the specific membrane resistance. These values were determined as described in the following: The soma diameter and dendrite length were measured from image stacks of GCs filled with Atto594 during dendritic recordings. In these experiments, the mean GC soma diameter was 5.9 µm and the mean length of GC dendrites, which were recorded from, was 20.7 µm (Table 1). The intracellular resistivity is similar across cell types and was set at previously estimated values from cerebellar GCs (100 Ωcm; Silver et al., 1992; Gabbiani et al., 1994; Cathala et al., 2003). The axon diameter was taken from the literature (0.18 µm, Sultan, 2000) and its length was set at 300 µm. The two remaining parameters—the diameter of the dendrites and the membrane resistance—are more difficult to measure. Since the diameter of GC dendrites is not exactly known and has a strong influence on the input resistance, we systematically varied the diameter of the dendrites in our GC model. We set the specific membrane resistance (*R*_m_) at a value that ensured that the model predicted our measured somatic *R_in_* of 492 MΩ for each dendrite diameter (Figure 3B, upper two graphs; cf. *Material and Methods*). We then calculated two parameters describing the electrotonic properties of GCs: the dendrite-to-soma conductance ratio,* ρ* (also referred to as dendritic dominance), and the electrotonic length of the dendrites, *L* (Rall, 1969). ρ increased with dendrite diameter (Figure 3B), corresponding to a larger contribution of the dendrites to *R_in_*. Also, *L* decreased with increasing dendrite diameter (Figure 3B), corresponding to increased electrotonic compactness and thus a convergence to a single compartment (which would have *L* = 0).

Based on these results, the measured dendritic *R_in_* was used to determine the average properties of our GCs. Therefore, we first determined the dendritic *R_in_* in our model as a function of dendrite diameter (Figure 3C, top graph; see *Material and Methods*). Comparison with the measured dendritic *R_in_* of 578.1 ± 64.9 MΩ revealed that our GCs are best characterized by a dendritic diameter of 0.52 µm. In our two-photon images, the patched GC dendrites had an average diameter of 0.69 ± 0.03 µm (*n* = 11, range 0.5–0.8 µm). Taking into account the limited spatial resolution of two-photon microscopy, these values seem consistent. According to the relations shown in Figure 3B, the comparison of the model prediction and the measured dendritic *R_in_* revealed an *R*_m_ of 1.4 kΩcm^2^, *ρ* of 1.23, and *L* of 0.15 (Figure 3C; see Table 1). These results and in particular the small electrotonic length of the dendrites demonstrate that GCs are electrotonically very compact.

We also used the measured membrane time constant as independent constraint in our simulations. To this end, we compared the measured membrane time constant with the one predicted by the model resulting in a graph comparable to Figure 3C (data not shown). This analysis yielded a dendrite diameter estimate of 0.69 µm, *R*_m_ of 1.7 kΩcm^2^, *ρ* of 1.64, and *L* of 0.12. These values are very similar to the approach based on *R_in_*, providing independent support for our conclusion of electronics compactness of GCs.

As described above, our measurements of *R_in_* might be influenced by the inward rectification present in GCs (cf. Figure 1). We therefore repeated the simulations as in Figures 3B,C with the higher *R_in_* values obtained from +20 pA current steps (cf. Figure 1G). The resulting estimates were dendrite diameter 0.52 µm, *R*_m_ = 3.7 kΩcm^2^, *ρ* = 1.23, and *L* = 0.10, again indicating that GCs can be considered as electrotonically very compact.

## Discussion

In this study, we established dendritic patch-clamp recordings from GC dendrites, which have a thin diameter of ~0.7 µm (Eccles et al., 1967). To the best of our knowledge, these are the thinnest dendrites recorded from. Dendritic recordings have been performed at other thin dendrites, such as the basal dendrites of layer 5 pyramidal neurons with a diameter of ~1.9 µm (Nevian et al., 2007), or dendrites of hippocampal basket cells with a diameter of ~1.4 µm (Hu et al., 2010; Nörenberg et al., 2010). We exploited this technique to directly investigate the passive electrical properties of GCs.

### Electrical Compactness of Cerebellar CGs

Previous studies using somatic recordings in rats indicated that GCs are electrotonically very compact (Silver et al., 1992; D’Angelo et al., 1993). In these two studies, the values for the dendrite-to-soma conductance ratio (*ρ*) were 0.98 and ≤0.5 (upper boundary), respectively. The electrotonic length of the dendrites (*L*) was determined as 0.05 and 0.04. These figures are slightly smaller than our estimates in mice of 1.23 for *ρ* and 0.15 for *L* (Figure 3; Table 1). Furthermore, the specific membrane resistance *R*_m_ was previously estimated as 16 kΩcm^2^ (Silver et al., 1992), whereas our estimate was 1.4 kΩcm^2^. Species and recording temperature differences could contribute to these discrepancies and may also explain why in our experiments at physiological temperature (35–37°C), *R_in_* was lower than previous estimates (D’Angelo et al., 1993, 1995; Brickley et al., 2001). In addition, the developmental state of the animals could be a reason, because pronounced changes in the morphological properties of GC and their membrane properties during development have previously been described (Cathala et al., 2003). For example, *R*_m_ was decreased from 9.2 kΩcm^2^ in P8 to 2.6 kΩcm^2^ in P39 mice (Cathala et al., 2003). Note, that our mice had an average age of P37, but previous studies used rats of age P10–P22 (Silver et al., 1992; D’Angelo et al., 1993). Nevertheless, the compactness of GCs was confirmed when using higher *R_in_* values for our analyses (see *Results*). Thus, our dendritic recordings strongly support the previous studies analyzing the electrotonic compactness of GCs. Furthermore, the low *R*_m_ of cerebellar GCs will contribute to a fast time course of EPSPs and facilitate rapid action potential initiation (Nörenberg et al., 2010). Consistent with the spatially uniform *R_in_* and electrotonic compactness, spontaneous EPSPs recorded at the soma and the dendrites were similar (Figure 2). Thus, our data indicate that cerebellar GCs are electrotonically extremely compact.

### Functional Implications

The electrotonic compactness allows GCs to rapidly and precisely integrate the fast EPSCs originating from mossy fiber activation (Silver et al., 1992; Cathala et al., 2005; Sargent et al., 2005) and to process high-frequency inputs (Saviane and Silver, 2006; Rancz et al., 2007; Ritzau-Jost et al., 2014). Furthermore, their compactness enables GCs to compare mossy fiber inputs independent of the distance of the synaptic site from the soma. Some less compact neurons with longer dendrites receive stronger inputs at distal parts of the dendrites (Magee and Cook, 2000) or express dendritic hyperpolarization-activated currents (Williams and Stuart, 2000) to counterbalance dendritic filtering of EPSPs. On the other hand, dendritic filtering might have the advantage to encode the spatial information of synaptic inputs (Rall, 1964). For GCs, however, this would not be of any benefit, because these neurons receive excitatory inputs only at the end of their dendrites. Furthermore, electrotonic compactness likely represents an important factor for the relay function of cerebellar GCs (Chadderton et al., 2004), which efficiently signal to postsynaptic stellate and Purkinje cells (Crowley et al., 2007; Valera et al., 2012), and thereby contribute to rapid cerebellar signaling (Blot and Barbour, 2014; Chen et al., 2014).

## Conclusion

In summary, our dendritic patch-clamp recordings demonstrate that dendrites of cerebellar GCs have a low dendritic dominance and short electrotonic length. Thus, GCs are electrotonically very compact, which seems ideally suited to rapidly process the high-frequency inputs arriving in the cerebellar cortex.

## Conflict of Interest Statement

The authors declare that the research was conducted in the absence of any commercial or financial relationships that could be construed as a potential conflict of interest.

## Footnotes

^1^http://www.neuromatic.thinkrandom.com
